# The Impact of Corporate Capital Structure on Financial Performance Based on Convolutional Neural Network

**DOI:** 10.1155/2022/5895560

**Published:** 2022-04-26

**Authors:** Yiheng Luo, Chenxi Jiang

**Affiliations:** ^1^Organization & Publicity Department, Xiang Nan University, Chenzhou 423000, China; ^2^School of Management and Economics, Xiang Nan University, Chenzhou 423000, China

## Abstract

Capital structure is an important indicator to measure the source, composition, and proportion of a company's equity and debit capital. It is not only related to the internal operating environment of listed companies but also related to the rights and obligations of shareholders and is closely related to the company's future development direction, decision-making bodies, and changes in governance structure. This study aims to study the impact of corporate capital structure on financial performance based on convolutional neural network. Based on the relevant theories of capital structure, by constructing a convolutional neural network model, taking a listed company as the research object, this study analyzes the company's capital structure, liabilities, and other financial conditions. Finally, it is concluded that short-term liabilities can meet the company's sustainable development and enhance the competitiveness of the industry, thereby increasing the company's operating income. However, a poor capital structure can negatively impact a company's finances. By improving the corporate governance structure of listed companies, strengthening the adjustment of the financing structure of listed companies, and strengthening the management of listed company's operating risks, the company's capital structure can be improved so that the company's financial situation can be sustainable and healthy.

## 1. Introduction

The purpose of establishing a company is to make a profit, and good corporate performance is the goal of the company's business activities. With the slowdown of our country's economic growth, my country's economy has entered a new normal [1, 2]. In the context of China's economic growth, how to maintain the continuous growth of corporate performance is a common problem faced by companies [3–6].

Some researchers have chosen to look at a sample of data from 3,500 SMEs in the UK, and ultimately, profitability, which is a proxy for firm performance, is independent of long-term gearing, which is a proxy for capital [7–10]. Some researchers put forward the priority financing theory based on the higher capital cost of equity financing than debt financing, but the large increase in debt scale is due to the internal capital of the company, and we think this is because we cannot meet the demand [11–15]. Therefore, debt levels and profitability are negatively correlated [16–18]. A researcher surveyed a data sample of listed companies in Malaysia and concluded the following. In highly commercialized situations, company performance is positively correlated with the possession and misuse of sensitive information. Performance is not affected by either [19, 20]. The two researchers conducted related research on the US banking industry as an example. Although its capital structure has a positive impact on corporate performance, its impact on capital structure is nonlinear [21, 22]. Some researchers have investigated the relationship between firm value and ownership structure and found that the relationship between the two is not significant. If there is only one major shareholder, there will be no increase in the enterprise value. For shareholders, the equity is relatively concentrated, and the value of the enterprise is easy to increase [23]. In conclusion, there are many factors that affect the relationship between capital structure and company performance, and different findings can be drawn by choosing different factors.

On the basis of consulting a large number of relevant references, combined with the relevant theories of capital structure and the impact mechanism of capital structure on corporate performance, this study takes listed companies in a certain industry as a research sample and constructs an econometric model and a convolutional neural network model. The model is empirically analyzed. With the help of the convolutional neural network model, the financial situation of the company is analyzed, and the impact of the company's capital structure on the financial situation is analyzed. It can be seen from the results that only by optimizing the company's capital structure, the company's financial situation can be sustainable and healthy.

## 2. Research on the Impact of Corporate Capital Structure on Financial Performance Based on Convolutional Neural Network

### 2.1. Theories Related to Capital Structure

#### 2.1.1. MM Theorem

MM theory is based on the relevant assumptions; one is that there is a perfect capital market. Second, the information is sufficient and complete. Third, all debts are safe. Fourth, there is a risk-free interest rate. Fifth, the impact of corporate income tax was not considered. Since the assumptions in the MM theorem are too strict to be established, the corporate income tax is added to the standard and the MM theorem is modified [24]. The model is

(1) $$\begin{array}{c} K_{\alpha }=K_{\beta }+M\times P. \end{array}$$

After adding personal income tax, the model is as follows:

(2) $$\begin{array}{c} K_{\alpha }=K_{\beta }+\left[1-\left(1-M_{\mu }\right)\left(1-M_{\lambda }\right)/\left(1-M_{\phi }\right)\right]\times P. \end{array}$$

#### 2.1.2. The Trade-Off Theory

In trade-off theory, companies have the greatest value. That is, there is an optimal capital structure. The theory holds that companies are worth more when bankruptcy costs can be offset by profits from the debt tax shield. According to the hedging theory, a company can use the debt tax shield to achieve the effect of a tax shield, thereby increasing the value of the company through debt management. However, the company's debt is subject to certain limits. Otherwise, continued debt growth increases the company's operating risk.

#### 2.1.3. Signal Transmission Theory

Investors can only obtain information from the company's public annual report and the materials that must be disclosed. If the company's asset-liability ratio has increased compared to the past, this is good news for investors, which means that the company's assets are increasing or its liabilities are decreasing, which reduces the investment risk of investors, so it will be invested favored [25]. If the company's debt ratio is reduced, the investment risk of investors will increase, so some investors will sell the company's stock, resulting in a decrease in the company's value.

#### 2.1.4. Agency Cost Theory

The agency cost theory believes that, with the prosperity of the market economy and the continuous expansion of the company scale, the owner cannot devote himself to the daily operation, and the profession of professional manager emerges as the times require. In order to meet the needs of the market economy, company owners are no longer responsible for the company's day-to-day operations, ownership and control are gradually separated, and new corporate forms emerge. The emergence of professional managers not only produces the relationship of representation but also conflicts among various stakeholders of the enterprise, as well as the cost of representation.

#### 2.1.5. Theory of Control Right

When Harris and Lavib looked at how corporate-led allocations affect capital structure, they argued that management has two ways of getting its own benefits. One is equity income and the other is control income. One theory is that the preference for control is pervasive among management, and if management wants to gain maximum control over the company, it changes the capital structure to achieve the goal of gaining more control [26]. At this time, the market value of the company has changed. If the management share is too large, the agency costs of the company will be high and the value of the company will decrease. According to this model, it is necessary to balance control and return on equity in order to optimize the allocation of the company's capital structure and maximize the company's value. The Stulz model focuses on the relationship between executive management and mergers and acquisitions. The Stulz model provides an optimal control structure by maximizing the expected return on investment. At the same time, Stulz believes that the company's stock price rise is not good news. The debt ratio is low and the possibility of acquisition is high. As such, Stulz argues if a company wants to acquire, it must have a debt level that maximizes the value of outside investors. Agion and Bolton added an unfinished contract theory to the model, analyzed capital structure, and discussed how to optimally distribute control between investors and traders. They argue that accounting for both transaction costs and incomplete contracts provides the best control when both investor profits and the firm's financial efficiency are maximized.

### 2.2. Influence Mechanism of Capital Structure on Company Performance

#### 2.2.1. The Impact of Financing Structure on Company Performance

Compared with equity financing, debt has the effect of tax avoidance, and its advantage is that it can reduce the tax paid to the company and improve the company's performance. When a company's debt income is greater than its cost of debt, operating leverage increases operating income and boosts the company's earnings. However, too much debt will increase the financial risk of the company, and even cause the capital chain to break, leading to business crisis or bankruptcy.

#### 2.2.2. The Impact of Debt Structure on Company Performance

The normal operation and development of the company is inseparable from debt. Only by increasing investment through debt can we quickly seize the market and win the competition. The cost of short-term debt is low, it will not have a great impact on the company, and the risk is controllable. However, short-term liabilities have higher requirements on cash flow, and the company must repay in a short period of time. When the company has sufficient cash flow, short-term debt is beneficial to the company's long-term development.

#### 2.2.3. The Impact of Ownership Structure on Company Performance

Ownership concentration is the core content of ownership structure, which has an important impact on corporate governance and corporate performance. Relative equity increases help address the “free-rider” problem, while increasing director oversight of major shareholders and reducing authorization costs. At the same time, major shareholders or their representatives have sufficient voting rights to participate in the company's business activities. In order to improve their own wealth, major shareholders actively improve the company's performance.

## 3. Experiment

### 3.1. Sample Selection and Data Sources

A sample survey of the financial data of companies in a certain industry was conducted from 2016 to 2019. Filter and clean data to remove incomplete data.

### 3.2. Variable Design

**Figure 1:**
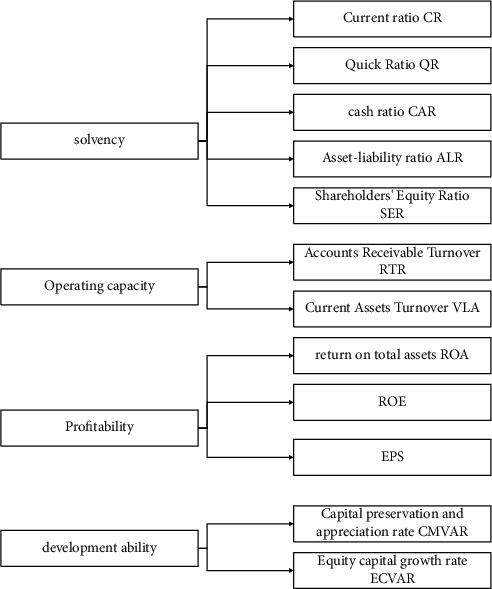
Company performance evaluation indicators.

Every company has its own unique business philosophy and approach, and when assessing a business's financial situation, it must be analyzed from multiple perspectives. Therefore, we adopted the method of multifactor analysis to analyze the correlation between multiple factors based on the original data and finally integrated them into a single indicator through screening.  Figure 1 shows various financial metrics.

### 3.3. Model Design

Analyze the relationship between the company's capital structure and finance by establishing the following model:

(3) $$\begin{array}{c} F=\rho ALR+\gamma MI+\sigma EPI+\omega , \end{array}$$

(4) $$\begin{array}{c} F=C+\rho CLR+\gamma MI+\sigma EPI. \end{array}$$

Among them, C is a constant term and *ρ*, *γ*, *σ*, and *ω* are the regression coefficients of the model.

### 3.4. Convolutional Neural Network Model Construction

The input layer is a 2D matrix, and the cells of each layer form a 3D matrix because “depth” is added to the congruence and concentration layers. The convolutional neural network is mainly composed of five structures: input layer, convolutional layer, centralized layer, fully connected layer, and output layer.

**Figure 2:**
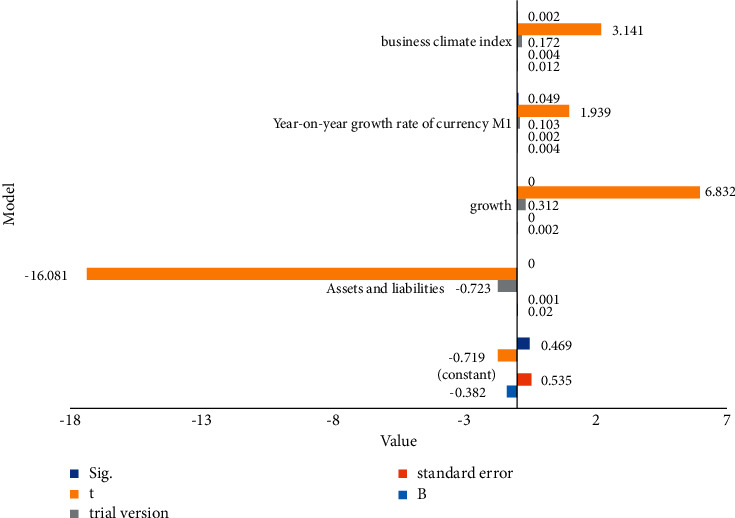
Coefficient.

With the help of the convolutional neural network model in Figure 2, we analyze the company's borrowing and financial status and then obtain the degree of influence of the company's capital structure on the company's financial performance.

#### 3.4.1. Input Layer

In a convolutional neural network, the input layer is the index data of any company, the input neurons are processed by many hidden layer neurons, and finally the listed company classification information is directly output to the output layer. During this time, the entire neural network undergoes significant weight adjustments.

#### 3.4.2. Convolutional Layer

Try using 1 ∗ 1, 3 ∗ 3, and 5 ∗ 5 as the kernel size. The depth of the convolution kernel is 1–6 levels. The simulation test results are shown in Table 1. When the size of the co-convergence score is 5 ∗ 5, the correct answer rate of the model is generally better than 1 ∗ 1 and 3 ∗ 3. Among them, when the convolution kernel size is 5 ∗ 5 and the depth is 6, the correct rate is the highest, which is 90.88%.

#### 3.4.3. Pooling Layer

The pooling layer has 6 feature maps of size 1 ∗ 1; each feature map is pooled from the convolution level by averaging 2 ∗ 2.

The pooling layer adopts the average concentration method, which integrates the feedback of all regions, does not change the output results, improves the robustness of the neural network, and strengthens the generalization of the convolutional neural network.

The average pooling can strengthen the convolutional neural network model and improve the analysis efficiency of the company's financial data.

#### 3.4.4. Fully Connected Layer

After the transfer function, the target classification is formalized as the loss function of the fully connected layer. The difference between the output value and the expected value is calculated and provided layer by layer through the backpropagation algorithm (BP algorithm), so the weight and polarization value of the convolution layer are continuously improved, and the output is a necessary network parameter.

#### 3.4.5. Output Layer

The OUTPUT level is also a fully connected level with a total of 5 units. The criterion is that if the output of a cell is 0 or close to 0, then the position of the cell in this layer is a number recognized by the network.

## 4. Discussion

### 4.1. Regression Analysis of the Impact of Capital Structure on Company Performance

Perform multiple regression in model (3).  Table 2 and Figure 2 show the regression results. As can be seen from these charts, ALR is positively correlated with CP. Listed companies in the industry have borrowed as much as 64 percent in recent years. According to hedging theory, the higher the value of ALR is, the more likely the company will go bankrupt, the more the company will pay its creditors, and the company's performance will continue to decline. The theory of priority lending believes that if maintaining an enterprise cannot cover the production and operation of the enterprise, then increasing the proportion of debt to borrowing is the first task of the enterprise.

**Figure 3:**
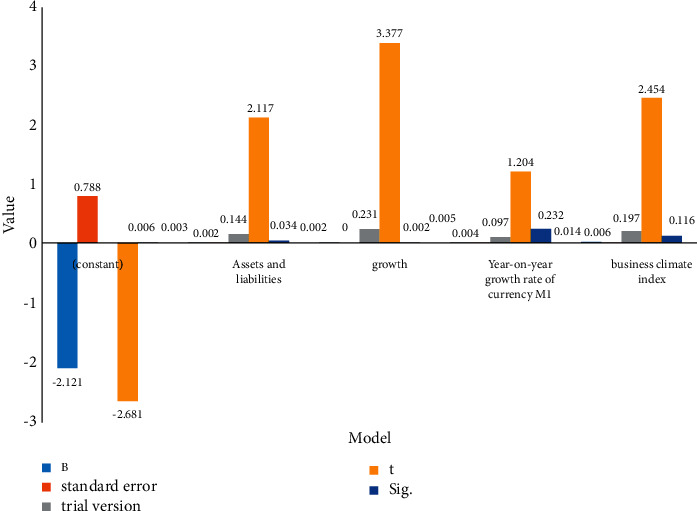
Coefficient.

Perform multiple regression on model (4).  Table 3 and Figure 3 show the regression results. As can be seen from the graphs, CLR is positively correlated with CP. Short-term debt has a strong incentive and restraint effect, which can reduce the possibility of company management using available cash, reduce investment in high-risk projects, and improve company performance.

### 4.2. Targeted Recommendations

#### 4.2.1. Improve the Corporate Governance Structure of Listed Companies

The shareholding structure of listed companies in my country is very concentrated, and there is an advantage phenomenon. In addition, according to the statistics of the business capability index, the management efficiency of the company is low, and the overall asset inflow/outflow rate is not ideal. This situation is mainly due to public companies, most of which are high-tech companies. However, with the listing, the company needs to change the original management concept and continuously improve the corporate governance system. In the case of information asymmetry in the process, only by maintaining the balance of interests of all parties can the subject effectively stimulate and restrict the mechanism and maximize value.

#### 4.2.2. Strengthen the Adjustment of the Financing Structure of Listed Companies

Capital structure management is an urgent management method, which requires enterprises not to blindly stick to the original capital structure and ignore the changes in capital structure caused by changes in environmental, economic, legal, political and cultural environments. The so-called emergency adjustment is to determine the unreasonable direction of the current capital structure according to changes in various situations. According to the current environment and the financial capital cost required by various financing methods, the company should find the best scope of capital structure suitable for business development. Companies can also regularly analyze and optimize the company's capital structure with the help of information tools such as convolutional neural networks.

#### 4.2.3. Strengthening the Management of Operational Risks of Listed Companies

Due to the imperfect information disclosure system, insufficient transparency of information disclosed in the Japanese capital market, and insufficient disclosure of information in the auditing industry, many listed companies have imperfect financial systems, and it is necessary to conduct credibility investigations. It is difficult to quantify a company's financial risk. Therefore, regardless of the government's views, it is mainly to improve relevant laws and systems, provide a legal basis for the development of listed companies, protect the interests of all participants, and make investors more confident. Entering the capital market, as far as the company itself is concerned, in order to strengthen risk management, the company strengthens the disclosure of financial and nonfinancial information and increases transparency, which is practical and effective for the company.

## 5. Conclusions

With the improvement of the economic level and the continuous growth and development of listed companies, some problems have gradually emerged, resulting in the effect of capacity utilization being less than expected, and the effect of equipment utilization being weakened and affected. Therefore, we will examine the relationship between a company's capital gains and financial performance, exploring the rationalization of its capital structure, reducing capital outflows, reducing the cost of capital, improving performance, taking high levels of financial risk, and improving the company's financial health. In addition, with the development of science and technology, companies can use information tools such as big data and convolutional neural networks to monitor the company's financial health in real time, optimize and adjust the company's capital structure, and avoid financial crises.

## Figures and Tables

**Table 1:** Comparison of the size and number of different convolution kernels.

Convolution kernel size	1 ∗ 1	1 ∗ 1	1 ∗ 1	1 ∗ 1	1 ∗ 1	1 ∗ 1
Number of convolution kernels	1	2	3	4	5	6
Accuracy	61.39%	70.36%	79.27%	61.43%	68.22%	75.98%
Convolution kernel size	3 ∗ 3	3 ∗ 3	3 ∗ 3	3 ∗ 3	3 ∗ 3	3 ∗ 3
Number of convolution kernels	1	2	3	4	5	6
Accuracy	72.83%	70.47%	77.31%	72.85%	77.36%	84.14%
Convolution kernel size	5 ∗ 5	5 ∗ 5	5 ∗ 5	5 ∗ 5	5 ∗ 5	5 ∗ 5
Number of convolution kernels	1	2	3	4	5	6
Accuracy	85.96%	84.29%	84.17%	81.75%	90.88%	80.09%

**Table 2:** ANOVA.

Model		Sum of square	df	Mean square	F	Sig.
3	Return	26.749	4	6.689	73.893	0.000
	Residual	18.377	201	0.088		
	Total	45.136	204			

**Table 3:** ANOVA.

Model		Sum of square	df	Mean square	F	Sig.
4	Return	4.256	3	1.059	5.277	0.000
	Residual	40.891	203	0.205		
	Total	45.137	204			

## Data Availability

The datasets used and/or analyzed during the current study are available from the corresponding author upon reasonable request.
